# Breast cancer incidence and early diagnosis in a family history risk and prevention clinic: 33-year experience in 14,311 women

**DOI:** 10.1007/s10549-021-06333-1

**Published:** 2021-07-26

**Authors:** D. Gareth Evans, Sacha J. Howell, Ashu Gandhi, Elke M. van Veen, Emma R. Woodward, James Harvey, Lester Barr, Andrew Wallace, Fiona Lalloo, Mary Wilson, Emma Hurley, Yit Lim, Anthony J. Maxwell, Elaine F. Harkness, Anthony Howell

**Affiliations:** 1 Clinical Genetics Service, Manchester Centre for Genomic Medicine, Manchester University Hospitals NHS Foundation Trust, Manchester, M13 9WL UK; 2 NW Genomic Laboratory Hub, Manchester Centre for Genomic Medicine, Manchester University Hospitals NHS Foundation Trust, Manchester, M13 9WL UK; 3 Division of Evolution and Genomic Sciences, Faculty of Biology, Manchester Academic Health Science Centre, School of Biological Sciences, Medicine and Health, University of Manchester, Manchester, UK; 4 Nightingale/Prevent Breast Cancer Centre, Wythenshawe Hospital, Manchester University NHS Foundation Trust, Manchester, M23 9LT UK; 5 Manchester Breast Centre, Manchester Cancer Research Centre, The Christie Hospital, Manchester, UK; 6 Division of Cancer Sciences, Faculty of Biology, Manchester Academic Health Science Centre, Medicine and Health, University of Manchester, Manchester, UK; 7 Division of Informatics, Imaging & Data Sciences, Faculty of Biology, School of Health Sciences, Medicine and Health, University of Manchester, Manchester, M13 9PT UK; 8 Department of Genetic Medicine, Manchester Academic Health Sciences Centre (MAHSC), St Mary’s Hospital, University of Manchester, Manchester, M13 9WL UK

**Keywords:** *BRCA1*, *BRCA2*, Breast cancer, Mammography, MRI, Screening

## Abstract

**Purpose:**

Women at increased familial breast cancer risk have been offered screening starting at an earlier age and increased frequency than national Screening Programmes for over 30 years. There are limited data on longer-term largescale implementation of this approach on cancer diagnosis.

**Methods:**

Women at our institution at ≥ 17% lifetime breast cancer risk have been offered enhanced screening with annual mammography starting at age 35 or 5-years younger than youngest affected relative, with upper age limit 50 for moderate and 60 for high-risk. Breast cancer pathology, stage and receptor status were assessed as well as survival from cancer diagnosis by Kaplan–Meier analysis.

**Results:**

Overall 14,311 women were seen and assessed for breast cancer risk, with 649 breast cancers occurring in 129,119 years follow up (post-prevalent annual incidence = 4.55/1000). Of 323/394 invasive breast cancers occurring whilst on enhanced screening, most were lymph-node negative (72.9%), T1 (≤ 20 mm, 73.2%) and stage-1 (61.4%), 126/394 stage2–4 (32%). 10-year breast cancer specific survival was 91.3% (95% CI 87.4–94.0) better than the 75.9% (95% CI 74.9–77.0) published for England in 2013–2017. As expected, survival was significantly better for women with screen detected cancers (*p* < 0.001). Ten-year survival was particularly good for those diagnosed ≤ 40 at 93.8% (*n* = 75; 95% CI 84.2–97.6). Women with lobular breast cancers had worse 10-year survival at 85.9% (95% CI 66.7–94.5). Breast cancer specific survival was good for 119 *BRCA1/2* carriers with 20-year survival in *BRCA1*:91.2% (95% CI 77.8–96.6) and 83.8% (62.6–93.5) for *BRCA2.*

**Conclusions:**

Targeted breast screening in women aged 30–60 years at increased familial risk is associated with good long-term survival that is substantially better than expected from population data.

**Supplementary Information:**

The online version contains supplementary material available at 10.1007/s10549-021-06333-1.

## Introduction

Breast cancer is the most common cancer in females with approximately 54,500 women diagnosed annually in the UK (2016) and remains the leading cause of premature death in women aged 30–60 years [1].

A family history risk and prevention clinic (FHRPC) to improve early detection and preventive approaches was established in Manchester in 1987 [2] and was the forerunner to other similar clinics across the UK and in Europe. In-house [3] and national [4] management guidelines were developed and, in, the UK, endorsed by a series of guidelines by the National Institute for Health and Care Excellence (NICE 2004, 2006, 2013, 2017) [5] in response to clinical developments in risk assessment, genetic testing, screening, preventive therapy and risk reducing surgery.

Risk was initially assessed by a modification of the Claus tables [6] and later using the Tyrer–Cuzick [7] and BOADICEA models [8, 9]. The cloning of *BRCA1* and *BRCA2* in 1994–1995 [10, 11] allowed predictive testing to identify which women had highest risk. Other high-penetrance breast cancer genes (lifetime-risk > 40%) including *TP53, PTEN, CDH1, STK11* and *PALB2* have also been identified, [12, 13] as well as moderate-risk genes (lifetime-risk 20–30%) *ATM* and *CHEK2* [12].

Annual breast screening was introduced from age 35/40–50 for women at moderate lifetime risk (17–29%), to age 60 for women at high risk (30% +) [5, 14–16] and MRI screening for those at very high risk [17–21]. Preventive therapy using tamoxifen and raloxifene was endorsed in the 2013 NICE guideline and anastrozole in 2017 [5, 21, 22]. The uptake of risk reducing breast surgery has gradually increased since its introduction in the UK in the early 1990’s [2].

We report here the outcome of risk assessment and surveillance in 14,311 women referred to the Manchester FHPRC clinic between 1987 and 2020, in particular, to assess breast cancer incidence and case-fatality. Outcomes from such a largescale systematic approach utilising family history to enhance early detection rates by mammography screening has not yet been published to our knowledge.

## Materials and methods

Women with a breast cancer family history, but unaffected personally, have been referred to the FHRPC at the Nightingale Centre Withington/Wythenshawe hospital since 1987. Their lifetime and residual risk of breast cancer has been assessed using questionnaire information on family history and standard risk factors using Claus tables and the Tyrer–Cuzick programme [2, 7–9, 23, 24]. Women were classified as being high-risk (lifetime-risk ≥ 30%), moderate-risk (lifetime-risk 17–29%) or average/population risk (lifetime-risk < 17%). Average-risk women were returned to primary care with reassurance and information on breast awareness and advised to start or continue screening from age 50. Moderate-risk and high-risk women were offered ‘enhanced’ surveillance [annual mammography and clinical breast examination (CBE)] starting at 35 years or 5 years younger than youngest affected relative (earliest age 30) with upper age limit 50 for moderate and 60 for high-risk. For high-risk women ‘enhanced’ surveillance continued at 12–18 monthly intervals from 50 to 60 years of age and started as young as age 30 (if *BRCA1/2* or youngest relative < 35). Women from families eligible for genetic testing (20% *BRCA1/2* likelihood 2004–2013, 10% likelihood > 2013) were referred to Genetics. Women with proven *BRCA1/2* pathogenic variants (PVs) in the family were offered targeted testing for their familial variant. In contrast, unaffected women (without breast cancer) with a significant family history have only been offered a full *BRCA1/2* screen since 2013 if an affected family member is unavailable and their a priori likelihood of a *BRCA1/2* PV is ≥ 10% [5]. *BRCA1/2* pathogenic variant (PV) carriers have been offered annual MRI screening aged 30–50 years since 2006 [5]. However, some aged 35–50 years had MRI screening through the MARIBS trial from 1997 [17]. MRI and mammography were performed simultaneously in MARIBS and from 2014 in the National Screening programme, but were offered at 6-monthly alternating intervals between 2006 and 2013. Women with lifetime breast cancer risks ≥ 25%, including *BRCA1/2* carriers, have had bilateral risk-reducing mastectomy (BRRM) discussions since 1994.

All FHRPC women seen from 1987 (including discharged) had assessment of vital (living/dead) and cancer status through the regional cancer registry and NHS systems in December-2012. Post 2012, deaths were notified to the clinic and breast cancer incidence was only assessed in those under ongoing surveillance. Women were censored for breast cancer incidence at: breast cancer diagnosis, BRRM, or death, if none, at last mammography (latest March 2020) or 01/12/2012. Data on all breast cancers occurring in the screening programme including interval cancers occurring within 18-months of last mammogram were collated. This included pathology [invasive–ductal, lobular, ductal carcinoma in situ (CIS)], tumour size, lymph node status and oestrogen (ER)/progesterone and HER2 receptor status. HER2 testing was only available from 2005. ER+ HER2− and triple negative breast cancer (TNBC) groups include HER2 untested from < 2005 as only 8.6% (6/70) and 7.3% (10/137) of subsequent tests on invasive ductal cancer for ER− and ER+ respectively were also HER2+ . Vital status was established on all breast cancer cases in April-2020 and causes of death confirmed from cancer records and death certification.

Breast cancers were defined as detected ‘on programme’ if they were diagnosed at screening episodes or within 18-months (interval cancers). Most women off programme were discharged to population breast screening on the 3-yearly basis NHS breast screening programme aged 50 if moderate-risk and 60 if high-risk.

In addition to clinical *BRCA1/2* (including *CHEK2* c.1100delC) testing where indicated by likelihood of a *BRCA1/2* PV, many families received testing of affected members through the Familial Breast Cancer Study (FBCS) and 1300 women (900 without breast cancer) consenting to the FHrisk study had testing of an extended panel of breast cancer genes through the BRIDGES study [25].

### Annual incidence rates

Annual incidence rates were calculated excluding cancers at prevalent screen. The *BRCA1* and *BRCA2* PV carrier groups were eligible for follow up from testing date or date of clinic entry which ever was later. PV carriers tested after breast cancer diagnosis were included in the untested group if their family PV was known at diagnosis or to the appropriate risk category at clinic entry if their family PV was identified after diagnosis. Likewise, those testing negative for their family *BRCA1/2* PV were eligible from date of negative test and excluded from prospective analysis as a PV carrier if testing was after diagnosis. All other women were grouped with their original risk category including the small group of 31 women with other known moderate/high risk gene PVs at entry. The 16 patients with neurofibromatosis-1 (NF1) were classified as moderate risk [26].

### Statistical methods

Breast cancer incidence was calculated excluding cancers detected on prevalence screen. Survival was assessed by Kaplan–Meier analysis and the log rank test to compare survival curves for categorical variables. Chi-squared tests were used to compare categorical variables. Differences in pathology variables in cancers on the screening programme used the high risk *BRCA1/2* negative group as the reference. *BRCA1* and *BRCA2* incidence and in those testing negative, was only assessed from date of mutation report. All *p* values were based on two-sided tests and were considered statistically significant if < 0.05. Analyses were performed using Stata version 14.

The study was approved by the Central Manchester Research Ethics Committee (10/H1008/24).

## Results

### Study population

**Fig. 1:**
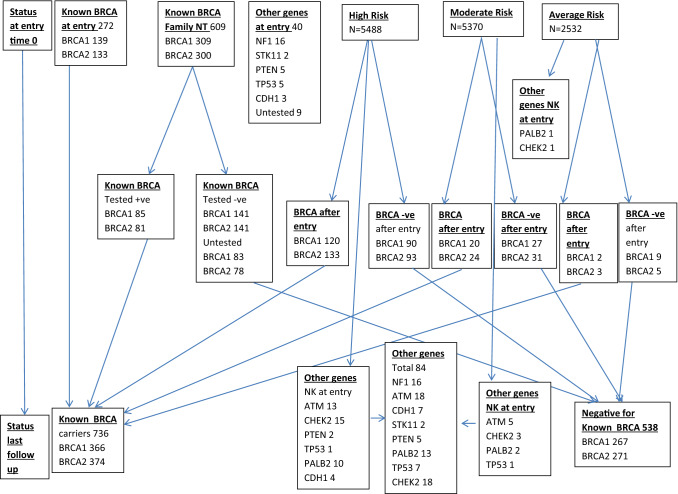
Flow diagram of Gene status and risk at entry and later. *NT* not tested, *NK* not known, *BRCA* −ve negative for known BRCA PV in family, *BRCA* +ve positive for known BRCA PV in family

A total of 14,311 women without breast cancer, born between 1920 and 2003 (median: 1966), had their risk of breast cancer assessed (Fig. 1). Age at entry ranged from 16 to 81 years (median = 39.9; IQR = 33.9–46.9). Detailed study population characteristics are described (Table 1) according to final known genetic status. 736 women (5.1%) have been identified as *BRCA* PV carriers (*BRCA1* = 364, *BRCA2* = 372) Table 1. 272 (37.0%) of these were referred into the FHRPC as known PV carriers unaffected by breast cancer. The remainder were identified after clinic entry (Fig. 1). As such 298/14,311 (2.1%) were identified as BRCA PV carriers with no known PV in the family at clinic entry. Overall BRCA testing in the individual woman or affected family member was completed in 4168 (29.1%) clinic attenders. Of 649 women with breast cancer, 539 (83.1%) had known BRCA status.

**Table 1:** Risk and gene PV status of all 14,311 women assessed at the Manchester FHRPC

Gene/risk	Number	Breast cancer	% BC (%)	RRM	% RRM (%)	Deceased	Median age at entry	IQR	Proportion of clinic (%)
*BRCA1*	366	77	21.04	121	33.24	20	35.7	31.0–42.5	2.54
*BRCA2*	374	78	20.86	101	27.15	19	36.9	31.9–45.0	2.60
*ATM*	18	9	50.0	0	0.00	0	41.7	37.9–45.0	0.11
*CDH1*	7	2	28.57	4	57.14	0	35.1	33.9–46.8	0.05
*STK11*	2	0	0.00	0	0.00	0	38.4		0.01
*PTEN*	5	2	40.00	1	20.00	1	35.8	35.3–36.5	0.03
*PALB2*	13	6	46.15	0	0.00	0	38.0	34.3–42.0	0.09
*CHEK2*	18	7	38.89	2	11.11	0	39.5	30.7–44.9	0.13
*NF1*	16	0	0.00	0	0.00	0	41.8	40.9–44.2	0.11
*TP53*	7	3	42.86	0	0.00	1	32.1	27.5–34.8	0.05
Negative for *BRCA1* in family	267	5	1.87	7	2.62	0	37.5	32.1–44.4	1.87
Negative for *BRCA2* in family	271	10	3.69	6	2.21	3	39.4	32.5–45.4	1.89
Untested for *BRCA1* in family	115	2	1.74	1	0.87	0	34.5	31.5–40.9	0.80
Untested for *BRCA2* in family	135	1	0.74	2	1.48	1	38.3	31.7–46.1	0.94
Negative for other actionable gene	13	0	0.00	2	15.38	0	36.2	34.0–42.3	0.09
Untested for other gene	10	0	0.00	0	0.00	0	35.1	28.2–42.3	0.07
*No known high or moderate risk gene in family or individual including untested*									
High	4939	217	4.39	171	3.46	69	39.7	34.7–47.0	34.53
Moderate risk	5234	181	3.46	37	0.71	73	40.3	34.2–46.5	36.59
Average/population risk	2501	48	1.92	0	0.00	82	41.5	33.9–34.7	17.48
Total	14311	649	4.51	455	3.18	269			100.00

*RRM* Risk reducing mastectomy, *IQR* Interquartile range, *BC* Breast cancer

### Follow-up time

There have been 129,119 women-years follow up with 649 breast cancers (588 post-prevalent), resulting in annual incidence of 4.55/1000. This excluded 45 prevalent asymptomatic screen detected cancers (0.31%) and a further sixteen women who developed symptomatic breast cancer between referral and clinic attendance. Therefore, there were 61 total prevalent cancers (0.43%–61/14311). Within the enhanced screening programme there were 63,972 years follow up, 349 (2.4%) women developed breast cancer following prevalence screen (incidence = 5.46/1000). 455 women (455/14311–3.2%) have undergone pre-symptomatic BRRM with seven occult breast cancers (1.5%) diagnosed at surgery. The remaining 239 cancers occurred after clinic discharge making a total of 255 (including 16 pre-prevalent scan) off programme. Breast cancer incidence by risk group is shown in Table 2. Incidence post prevalence in *BRCA1* was 1.73% and in *BRCA2* 1.55% annually from date of mutation report. The low rate in untested women reflects those at 25% risk and very young women prior to testing. Carriers of known PVs in other genes were included in the lifetime risk category known at entry due to low numbers.

**Table 2:** Incidence rates for breast cancer by *BRCA1/2* and risk group

	Number	Follow up	BC	BC annual rate (%)	Prevalent	% Prevalent (%)
*BRCA1*^a^	309	1738.4	30	1.73	2	0.65
*BRCA2*^a^	312	1811.3	28	1.55	2	0.64
moderate in screening	5293	28087.3	100	0.36	19	0.36
High risk in screening	5129	30392.3	192	0.63	29	0.57
Moderate off screening		22091.6	83	0.38	N/a	
High risk off screening		13298.5	81	0.61	N/a	
Average	2509	23924.8	49	0.20	1	0.04
Negative for family *BRCA2*	218	1459.0	6	0.41	0	
Negative for family *BRCA1*	194	1379.2	4	0.29	0	
Untested for family *BRCA1* +	167	2390.3	7	0.29	3	1.80
Untested for family *BRCA2* +	180	2546.9	8	0.31	5	2.78
	14311	129119.5	588		61	

^a^Includes 31 tested on research basis who have not had clinical testing including 7 *BRCA1/2* with breast cancer + includes women tested after censor and follow up in women who later tested positive or negative but these numbers not included in total women

*BC* breast cancer

### Cancers

The age and known PV carrier status of the women with breast cancers are shown in Table 3. Cancer pathology on the FHRPC enhanced screening programme is shown in Table 4. *BRCA1*-related breast cancers were more likely grade 3 and oestrogen receptor negative (ER−) than cases in the high-risk BRCA negative cohort as expected (*p* < 0.0001 for both). Tumours from women who tested BRCA PV negative in the high-risk cohort were more likely to be grade 1 than both *BRCA1* (*p* < 0.001) and *BRCA2* (*p* = 0.04) tumours. Only 36/394 (9%) women with cancers in the enhanced screening programme had no genetic testing compared with 84/255 (32.9%) of those off programme. Of the cancers in the enhanced screening programme 70 (17.9%) were CIS with a higher proportion seen on prevalent screen (33.3%—Supplementary Table S1). The majority of invasive cancers were lymph node negative (72.9%), small (≤ 20 mm–73.2%) and stage-1 (61.4%). *BRCA1*/*BRCA2* PV associated cancers were smaller overall with 75.0% and 85.4% being ≤ 20 mm respectively, potentially reflecting MRI screening in these groups. As screening was not universal < 35 years we assessed the incidence of breast cancers aged 30–35 years in those with or without a relative diagnosed at < 35 years. Of 1700 with a relative diagnosed < 35, 740 were seen aged < 35 and 7 (0.95%) diagnosed aged 30–35. In contrast of 12,611 without such family history 15/3362 (0.45%) seen < 35 developed breast cancer aged 30–35 (*p* = 0.098).

**Table 3:** Gene status in women with breast cancer with age at diagnosis and whether on or off programme

Gene/risk	Total breast cancer	On screening program	Median age	Range	IQR	Died	% Died (%)	Off screening programme	Median age	Range	IQR	Died	% Died (%)
*BRCA1*	77	60	43.2	29.5–61.3	36.6–48.7	8	13.79	17	42.5	27.7–66.4	38.0–53.5	3	17.65
*BRCA2*	78	59	46.9	28.7–77.1	39.3–52.0	8	13.79	19	54.2	40.4–71.4	48.7–60.3	3	15.79
*ATM*	9	8	47.4	39.8–53.4	46.0–52.5	1	16.67	1	41.6	41.0		0	0.00
*CDH1*	2	1	50.3			1	100.00	1	51.3			1	100.00
*PTEN*	2	2	39.9	38.4–41.4		0	0.00	0				0	
*PALB2*	6	6	44.7	41.6–46.5	44.5–45.4	1	16.67	0				0	0.00
CHEK2	7	4	43.4	35.6–46.1	36.9–44.1	0	0.00	3	64.4	54.4–74.5		3	100.00
TP53	3	1	24.1	24.1		0	0.00	2	32.4	28.9–35.9		1	50.00
Negative for family *BRCA1*	5	3	54.3	47.4–56.9		0	0.00	2	51.2			0	0.00
Negative for family *BRCA2*	10	4	54.1	49.7–59.3	51.2–57.3	1	25.00	6	52.2	44.5–53.1	49.8–52.6	0	0.00
Untested *BRCA1*	2							2	53.8	41.5–66.0		1	50.00
Untested *BRCA2*	1							1	56.5			0	0.00
Average risk negative *BRCA1/2*	22	3	55.0	48.3–64.9		3	100.00	19	55.9	31.4–66.6	51.6–58.3	4	21.05
Moderate risk negative *BRCA1/2*	121	78	47.9	34.3–65.5	44.5–52.1	14	17.50	43	56.2	30.2–78.1	48.5–65.4	5	11.63
High risk negative *BRCA1/2*	176	131	49.7	29.6–66.6	44.9–54.8	14	10.53	45	54.7	32.1–79.2	50.1–67.3	9	20.00
Average risk no testing	26	0				0		26	55.7	41.3–86.6	51.8–63.0	11	42.31
Moderate risk no testing	61	17	48.4	34.4–64.0	46.4–53.4	1	5.88	44	55.3	27.6–71.1	51.1–62.6	10	22.73
High risk no testing	41	17	45.9	33.0–63.0	44.8–54.8	2	11.11	24	56.7	35.5–71.1	47.7–64.6	2	8.33
Total	649	394				54	13.81	255				53	20.78

*IQR* Interquartile range, *BC* Breast cancer

**Table 4:** Pathology details by genetic testing and risk group of breast cancers identified on the screening programme

Gene/risk	*BRCA1*	*BRCA2*	*ATM*	*CDH1*	*PTEN*	*PALB2*	*CHEK2*	*TP53*	Negative for family BRCA mutation	Average risk	Moderate risk negative *BRCA1/2*	High risk negative *BRCA1/2*	Moderate risk no testing	High risk no testing	Total
On breast screening programme	60	59	8	1	2	6	4	1	7	3	78	131	17	17	394
Timing of cancer diagnosis															
Prevalent	5	7	0	0	0	0	1	0	1	1	17	9	3	1	45
Incident	37	42	8	1	2	4	2	1	6	0	43	87	10	13	255
Interval % interval (%)	18 30.0	10 16.9	0 0.0	0 0.0	0 0.0	2 33.3	1 25.0	0 0.0	0 0.0	2 66.7	18 23.1	35 26.7	4 23.5	317.6	94 23.9
Interval at BRRM	2	2		1			1					1			7
Type of cancer															
IDC Grade 1	0	3	0	0	0		1	0	0	1	10	26	2	1	45
*p* value Grade 1	< 0.0001	0.004									0.18	Reference			
IDC Grade 2	7	18	2	0	1	2	1	0	2	0	25	35	5	6	102
IDC Grade 3 % Grade 3 (%)	48 80.0	21 35.6	1 12.5	0 0.0	0 0.0	4 66.7	1 25.0	1 100.0	3 42.9	0 0.0	25 32.1	32 24.4	5 29.4	4 23.5	143
Grade 3 vs high risk no PV*	< 0.0001	0.24									0.73	Reference			
ILC	0	3	1	1	0	0	0	0	0	2	5	17	1	2	32
*p* value*	< 0.0001	0.1									0.16	Reference			
CIS % CIS (%)	5 8.3	14 23.7	4 50.0	0 0.0	1 50.0	0 0.0	1 25.0	0 0.0	2 28.6	0 0.0	13 16.7	21 16.0	4 23.5	4 22.2	70 17.9
*p* value*	0.18	0.08									1.0	Reference			
Cancer characteristics															
LN negative in invasive % LN^0^ (%)	40 72.7	34 75.6	4 100.0	1 100.0	1 100.0	4 66.7	3 100.0	1 100.0	3 60.0	1 33.3	46 70.8	80 72.7	8 53.3	11 84.6	234 72.9
Stage 1 % of invasive (%)	35 63.6	30 66.7	250.0	0 0.0	0 0.0	2 33.3	3 100.0	1 100.0	3 60.0	1 33.3	36 55.4	69 62.7	7 46.7	7 53.8	197 61.4
Invasive ≤ 20 mm %	39 73.6	36 83.7	1 33.3	0 0.0	0 0.0	2 33.3	3 100.0	1 100.0	4 80.0	1 33.3	48 73.8	82 74.5	9 60.0	7 53.8	235 73.2
ER-	42	14	0	0	0	1	0	0	0	1	22	16	3	2	101
	< 0.0001	0.05									0.004	Reference			
HER2+	0	2	0	0	0		0	0	0	0	7	5	1	1	16

*BRRM* bilateral risk reducing mastectomy, *IDC* invasive ductal carcinoma, *ILC* invasive lobular carcinoma, *CIS* carcinoma in situ, *LN* lymph node, *PV* Pathogenic variant

*p values were compared with high risk PV negative for *BRCA1, BRCA2* and moderate only due to numbers

#### Clinical Breast Examination (CBE)

Sixteen breast cancers (4%) were initially identified as mammographically occult, but detected at the routine screen by CBE. Diagnoses were made after ultrasound guided biopsy with 9/16 at stage-1 and 11 ≤ 20 mm. 36 cancers were palpable at the mammogram appointment including 1/38 on the MRI program that was missed on previous MRI, but detected on alternating mammography (size = 29 mm).

##### MRI screened

Thirty-eight women were diagnosed with breast cancer on the MRI programme (*BRCA1* = 21; *BRCA2* = 16; *TP53* = 1). There were four occult cancers found at BRRM: three DCIS 2–17 mm and an invasive 13 mm tumor. Of those detected by screening 31/34 (91%) were ≤ 20 mm (DCIS = 1) and 18/34 (53%) ≤ 10 mm. Overall 7/38 (18.4%) were DCIS, with only two stage-2 cancers including the only case with lymph node involvement (size = 29 mm-palpable).

##### Cancer deaths

Overall deaths were lower in women screened on the enhanced-programme (13.8%) compared with off-programme (20.8%) (Table 3), although off-programme women were older. Of the deaths with *BRCA1/2* PVs, 7/16 were unrelated to breast cancer; *BRCA1-*4/8 [ovarian (*n* = 2), carcinosarcoma uterus (*n* = 1), pancreatic (*n* = 1)], *BRCA2-*3/8 (ovarian, lung cancer, old-age). Only one each *BRCA1* and *BRCA2* deaths in carriers were breast cancer related in women on MRI screening (2/38). In total 34/54(63%) deaths in women with cancers detected in the enhanced screening programme were breast cancer related. There were two cancer deaths in women undergoing BRRM who had breast cancer (one breast, one pancreatic) (0.44%) as well four non-cancer deaths for a total of 6/455 (1.3%), compared to 274 total deaths in women not undergoing BRRM (2%).

##### Cancer survival

**Fig. 2:**
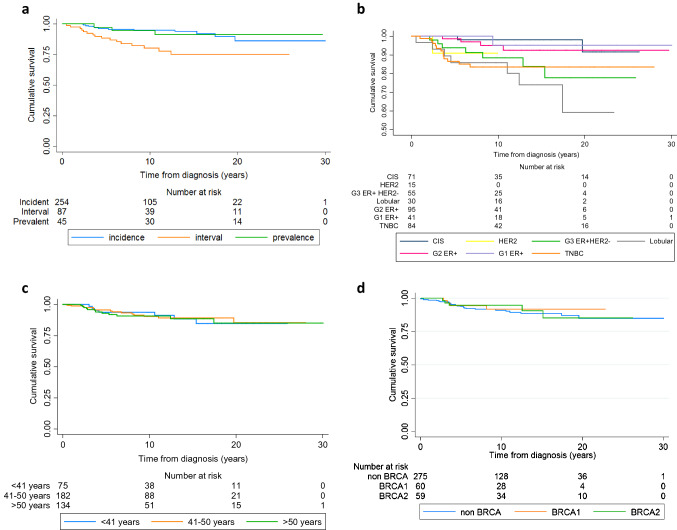
**a** Survival by presentation (interval, incident, prevalent)—breast cancer deaths. Incidence vs interval (*p* < 0.001), prevalence vs incidence (*p* = 0.052). **b** Survival by pathology type—breast cancer deaths. TNBC vs G1 ER+ (*p* = 0.04), G2 ER+ (*p* = 0.03), CIS (*p* = 0.01); G1 ER+ vs lobular (*p* = 0.015); G2 ER+ vs lobular (*p* = 0.006); lobular vs CIS (*p* < 0.001); G3 ER+ HER2− vs CIS (*p* = 0.019). *TNBC* triple negative breast cancer, *CIS* carcinoma in situ, *G* grade, *ER* estrogen receptor, *HER2* human epidermal growth factor receptor 2. **c** Survival by age group—breast cancer deaths. **d** Kaplan–Meier breast cancer specific survival curves comparing *BRCA1, BRCA2,* and non-BRCA affected women

The 10-year breast cancer specific survival in women in the enhanced screening programme combined from prevalent, incident and interval cancers was 91.3% (95% CI 87.4–94.0). Breast cancer deaths, as expected, were more frequent in women with symptomatic interval cancers (Supplementary Table S1, Fig. 2a). Incident screen detected 10-year survival was 91.9% (95% CI 86.7–95.1) vs interval 80.2% (95% CI 68.6–87.9) (*p* < 0.001); prevalence screen survival 94.5% (95% CI 79.8–98.6) vs incidence screen (*p* = 0.052). The pathologies with the highest proportion of breast cancer deaths were lobular [23.3%, 10-year survival = 85.9% (95% CI 66.7–94.5)] triple negative [14.3%; 10-year-survival = 83.5% (95% CI 72.7–90.3)] and high-grade ER+ HER2− cancers [13.0%; 10-year-survival = 88.5% (95% CI 74.3–95.1)] although numbers in each group were relatively small limiting statistical comparison. As expected, the lowest proportion of breast cancer specific deaths was noted in those with grade-1 tumours [2.4%; 10-year-survival = 95.5% (95% CI 70.7–99.3)] and CIS [2.8%; 10-year-survival = 98.2% (95% CI 87.6–99.7)], although breast cancer specific survival was also excellent in grade-2 ER + HER2- breast cancer [10-year-survival = 95.1% (95% CI 85.3–98.4)]. Nearly all triple negative breast cancer deaths occurred in the first 5-years (Fig. 2b).

Although overall survival was worse in those diagnosed > 50 years (10-year = 83.5% ≤  40 years 10-year = 93.5%, *p* = 0.04; 41–50 years 10-year = 88.8%, *p* = 0.025—Supplementary Figure S1), breast cancer specific survival was virtually identical for all age groups, with 10-year ≤ 40 years survival 93.8% (95% CI 84.2–97.6—Supplementary Table S1; Fig. 2c). For the ≤ 40 years group with invasive breast cancer (*n* = 58), 5, 10 and 20-year overall survival was 92.2% (80.5–97.0), 92.2% (80.5–97.0) and 79.9% (59.9–90.6). Survival was not significantly different between *BRCA1, BRCA2* and non-BRCA carriers on enhanced screening (Fig. 2d) with 20-year breast cancer specific survival particularly good in 60 *BRCA1* carriers at 91.5% (78.5–96.8) compared to 59 *BRCA2* at 85.1% (64.1–94.3) and 275 non-BRCA 84.7% (76.5–90.3). The *BRCA2* survival curve crossed over *BRCA1* after 10 years. Only 51 BRCA carriers were aware of their status at breast cancer diagnosis. Kaplan–Meier curves comparing BRCA PV carriers who knew their status at diagnosis versus those who did not and BRCA carriers who had MRI versus those who only had mammography are shown in Supplementary Figures S2 and S3. Survival in the known carriers and MRI screened (90.6%, 95% CI 80.3–95.7%) and 90.1% (95% CI 62.6– 97.7%) 10-year survival respectively, but this was not significantly better than the controls who did not know their status (94.8%; 95% CI 68.0–93.2%) and those not undergoing MRI (87.0%: 95% CI 80.6–94.8%). We also carried out a time dependency analysis and this did not show any advantage to knowing the BRCA status (Supplementary Figure S4). Overall 20-year cumulative risk of breast cancer was 9.7% (95% CI 8.9–10.7%—Supplementary Figure S5).

Five and 10-year breast cancer specific survival in those with breast cancer detected on programme vs off programme screening was:5-year 94.1% (95% CI 91.0–96.1) vs 94.3% (95% CI 90.5–96.6) and 10-year 91.0% (95% CI 87.2–93.7) vs 90.4% (95% CI 85.7–93.4) respectively. Overall 20-year survival in the cohort for all women for breast cancer specific survival was 98.8% (95% CI 98.3–99.1%) and for known *BRCA1* carriers (*n* = 365) = 96.1% (95% CI 90.5–98.4%) and *BRCA2* (*n* = 376) = 91.5% (95% CI 78.6–96.8%). The latter should be regarded as an underestimate as not all women had undergone *BRCA1/2* testing.

## Discussion

The current study is, to our knowledge, the largest study on systematic local approaches to breast cancer risk assessment and surveillance. In total 649 (4.5%) of 14,311 women developed breast cancer with the majority [394 (62%)] detected on enhanced screening. The majority of breast cancers were detected at stages 0/1 (270/394–68.5%) with only 94/394 (23.5%) interval cancers, seven of which were asymptomatic at BRRM. There were expected breast cancer associations with *BRCA1* grade-3 ER− HER2− [27], significantly more frequent than high-risk BRCA-negative group (*p* < 0.0001). Pure CIS was less frequent in *BRCA1* and more frequent in *BRCA2* [27]. There appears to be a stronger signal for low grade breast cancer (28%) in those testing negative for PVs in high-risk genes suggesting a potential feature of yet to be discovered moderate/high-risk genes.

We have reported a mean annual rate of incident prospective breast cancers in *BRCA1/2* PV carriers of 1.6% (1.55% *BRCA2*, 1.73% *BRCA1*) [28], consistent with currently published 69–72% risks by age 80 years [29, 30] when extrapolated over a 50-year risk period. This study also provides support for the current NICE recommended annual MRI screening surveillance strategy [5] as there was only one death in a *BRCA2* carrier among MRI screened women. This continues to provide efficacy evidence for MRI as an alternative to BRRM [31], although 83% 20-year survival will still not convince many *BRCA2* carriers. Indeed, nearly half the deaths (44%) in *BRCA1/2* PV carriers with breast cancers were due to other cancers (6/16) or old age (1/16). A study of 491 women with a germline BRCA mutation, who were annually screened with MRI and mammography for a median of 12.7 years found incidence breast cancer was 2% annually. There were four breast cancer-related deaths among 91 who developed breast cancer [32].

Breast cancer specific survival was excellent with 10-year survival rates of 91.3% (95% CI 87.4–94.0) noticeably higher than current 10-year breast cancer survival in England of all women presenting with primary breast cancer of 75.9% (95% CI 74.9–77.0; 2013–2017 data) [33]. Of particular note is that the 10-year invasive breast cancer survival ≤ 40 years of 92.2% (95% CI 80.5–97.0) had lower 95% CI above the UK population based POSH (Prospective-study-of-Outcomes-in-Sporadic-versus-Hereditary-breast-cancer) trial. This trial consisted of women presenting with primary breast cancer ≤ 40 years between 2000 and 2008 [34]. The study found 10-year survival of 73·4% (67·4–78·5) for *BRCA1/2* vs 70·1% (67·7–72·3) for non BRCA breast cancer compared to the lower 95% CI of 86.9% in our population. Indeed, the Kaplan–Meier curves continued to drop towards 50% by 15 years in POSH [34] far below the 20-year survival from our study of 85.3% (77.1–90.7), and indeed the lower 95% CI. Thus, even allowing ~ 18-months lead-time [16] survival of women with invasive cancers ≤ 40 who undergo annual screening is likely significantly better than unscreened women as suggested by the FH02 study [16].

As expected, women with interval breast cancers had higher mortality. Most presented within 12-months with known poorer survival [35]. Interestingly stage 2 or higher breast cancer was not different between incident and prevalent cases (27.6% vs 26.7%) among the screened population and this is reflected by similar survival rates. This is partly explained by the higher rates of DCIS (33% vs 20.5%). Although women presenting with triple negative cancers had relatively low 5-year survival, at 10–15 years this was no worse than for those with high-grade ER+ HER2− breast cancer. Interestingly, invasive lobular breast cancer, known to have higher interval cancer rates [29] presumably poorer mammographic sensitivity, was associated with the worst survival. Individuals with a higher risk of lobular cancer including those with *CDH1* pathogenic variants, LCIS or lobular breast cancer family history should be considered for MRI breast screening. A small number of cancers (4%) were detected only on CBE with a minority of the screen detected cancers also detected. We have previously highlighted this, but whether this is a cost-effective strategy despite the small size of most of these cancers will require further work [36]. We have also shown that use of a polygenic risk score from multiple Single Nucleotide Polymorphisms is accurate in reclassifying risk and will be a useful tool in targeting family history-based screening in future [14].

There are some limitations to the present study. Genetic testing was only carried out in a minority of the screened population although it was performed on the great majority of enhanced programme breast cancers (91%) and assessment of incidence rates based on gene testing was not the primary study aim. Most women with breast cancers had panel testing, allowing extrapolation of likely frequencies of other common familial genes (*ATM, CHEK2, PALB2*). We do not have follow up for all women after December-2012, but were able to check vital status and cause of death for all women with breast cancer.

In conclusion, the present study has demonstrated good survival from family history based enhanced-screening approach over a 33-year period. Overall and breast cancer specific survival is very good and substantially better than would be expected from population statistics and especially ≤ 40 years who would not otherwise qualify for screening. MRI screening is of benefit to *BRCA1/2* carriers and could also be utilised in those at high risk of lobular cancer who are otherwise less well served by mammography.

## Supplementary Information

Below is the link to the electronic supplementary material.

Supplementary file1 (DOCX 43 KB)

Supplementary file2 (DOCX 89 KB)

## Data Availability

Raw data available on request.
