# Supplementary material for: Breast cancer incidence and early diagnosis in a family history risk and prevention clinic: 33-year experience in 14,311 women
Source: Breast Cancer Res Treat. 2021 Jul 26;189(3):677–87. doi: 10.1007/s10549-021-06333-1 (PMC8505388; doi:10.1007/s10549-021-06333-1)
Supplement: Supplementary file 1 — Supplementary file1 (DOCX 43 KB) [file 10549_2021_6333_MOESM1_ESM.docx]

**Supplementary Figure 1: Survival by age group - all deaths**

Age group 1 (≤40) vs 3 (>50) ( p=0.04), 2 (41-50) vs 3 (p=0.025)

**Supplementary Figure 2: Survival from diagnosis to death in BRCA carriers by mutation status at diagnosis**

| Known mutation at diagnosis | Survival | 95% CI |
| --- | --- | --- |
| No (68), deaths (10) |  |  |
| 5 | 92.1 | 82.5 – 96.7 |
| 10 | 90.6 | 80.3 – 95.7 |
| 20 | 76.1 | 57.4 – 87.4 |
| Yes (51), deaths (6) |  |  |
| 5 | 91.4 | 78.8 – 96.7 |
| 10 | 84.8 | 68.0 – 93.2 |
| 20 | 84.8 | 68.0 – 93.2 |

Log rank chi2 = 0.13, p = 0.72

**Supplementary figure 3: Survival from diagnosis to death in BRCA carriers by MRI status**

| MRI | Survival | 95% CI |
| --- | --- | --- |
| No (81), deaths (14) |  |  |
| 5 | 89.7 | 80.6 – 94.8 |
| 10 | 87.0 | 77.1 – 92.9 |
| 20 | 73.7 | 56.6 – 84.9 |
| Yes (38), deaths (2) |  |  |
| 5 | 97.1 | 80.9 – 99.6 |
| 10 | 90.1 | 62.6 – 97.7 |
| 20 | - | - |

Log rank chi2 = 1.28, p=0.26

**Supplementary figure 4:Survival from diagnosis to breast cancer death by BRCA status (time dependent i.e. those with unknown status originally classed as non-BRCA to date of individual mutation testing and as BRCA1/2 thereafter if positive test)**

| BRCA status | Survival | 95% CI |
| --- | --- | --- |
| Non BRCA (deaths 25) |  |  |
| 5 | 94.1 | 90.4 – 96.4 |
| 10 | 91.4 | 86.9 – 94.4 |
| 20 | 85.3 | 77.1 – 90.7 |
| BRCA1 (deaths 4) |  |  |
| 5 | 94.0 | 82.5 – 98.0 |
| 10 | 91.2 | 77.8 – 96.6 |
| 20 | 91.2 | 77.8 – 96.6 |
| BRCA2 (deaths 5) |  |  |
| 5 | 93.7 | 81.6 – 97.9 |
| 10 | 93.7 | 81.6 – 97.9 |
| 20 | 83.8 | 62.6 – 93.5 |

p>0.05 for all comparisons

**Supplementary figure 5:** Kaplan-Meier cumulative risk (from date of entry to censored date) of breast cancer (censored reason = breast cancer) in 14311 women attending the FH clinic

|  | Cumulative risk (95% CI) |
| --- | --- |
| 5-year | 0.021 (0.019 – 0.024) |
| 10-year | 0.045 (0.041 - 0.050) |
| 15-year | 0.072 (0.066 – 0.078) |
| 20-year | 0.097 (0.089 – 0.107) |
| 25-year | 0.141 (0.118 – 0.168) |
| 30-year | 0.198 (0.142 – 0.271) |
