# Supplementary material for: Breast cancer incidence and early diagnosis in a family history risk and prevention clinic: 33-year experience in 14,311 women
Source: Breast Cancer Res Treat. 2021 Jul 26;189(3):677–87. doi: 10.1007/s10549-021-06333-1 (PMC8505388; doi:10.1007/s10549-021-06333-1)
Supplement: Supplementary file 2 — Supplementary file2 (DOCX 89 KB) [file 10549_2021_6333_MOESM2_ESM.docx]

**Supplementary Table 1:** Carcinoma *in situ* (CIS), Stage 2 invasive or greater pathology and deaths among breast cancers in the screening programme. Survival is stated for each row heading rather than stage 2+.

|  | Stage 2+ | % | CIS | % CIS | Deaths | Total number of women | % died | BC specific deaths | | 5-year survival | 10-year survival | BC specific 5 year survival | BC specific 10 year survival |
| --- | --- | --- | --- | --- | --- | --- | --- | --- | --- | --- | --- | --- | --- |
| Time of diagnosis | | | | | | | | | | | | | |
| Interval symptomatic | 46 | 52.9% | 2 | 2.4% | 22 | 87 | 25.3% | 16 | 18.8% | 83.7 (73.6 – 90.2) | 74.6 (62.9 – 83.1) | 88.4 (78.8 – 93.8) | 80.2 (68.6 – 87.9) |
| Interval RRM | 0 | 0.0% | 2 | 28.6% | 1 | 7 | 14.3% | 1 | 14.3% | 80.0 (20.4 – 96.9) | 80.0 (20.4 – 96.9) | 80.0 (20.4 – 96.9) | 80.0 (20.4 – 96.9) |
| Incident | 70 | 27.5% | 52 | 20.4% | 22 | 255 | 8.6% | 14 | 5.5% | 95.3 (91.4 – 97.4) | 91.9 (86.7 – 95.1) | 96.3 (92.7 – 98.1) | 95.0 (90.9 – 97.3) |
| Prevalent | 12 | 26.7% | 16 | 33.3% | 8 | 45 | 17.8% | 3 | 6.7% | 97.3 (82.3 – 99.6) | 94.5 (79.8 – 98.6) | 97.3 (82.3 -99.6) | 94.5 (79.8 – 98.6) |
| Total | 128 | 32.5% | 71 | 18.2% | 53 | 394 | 13.5% | 34 | 8.7% | 92.7 (89.3 – 95.0) | 88.0 (83.7 – 91.2) | 94.4 (91.3 – 96.4) | 91.3 (87.4 – 94.0) |
| Age at diagnosis | | | | | | | | | | | | | |
| ≤40 years | 17 | 21.8% | 17 | 21.8% | 7 | 78 | 9.0% | 7 | 9.0% | 93.8 (84.2 – 97.6) | 93.8 (84.2 – 97.6) | 93.8 (84.2 – 97.6) | 93.8 (84.2 – 97.6) |
| 41-50 years | 63 | 34.6% | 37 | 20.3% | 20 | 182 | 11.0% | 15 | 6.6% | 95.6 (91.0 – 97.9) | 88.8 (81.9 – 93.2) | 95.6 (91.0 – 97.9) | 90.5 (84.0 – 94.4) |
| >50 years | 48 | 35.1% | 17 | 12.7% | 27 | 134 | 19.4% | 12 | 11.2% | 88.2 (80.9 – 92.9) | 83.5 (74.8 – 89.4) | 93.1 (86.6 – 96.5) | 90.9 (83.6 – 95.0) |
| Total | 128 | 32.5% | 71 | 18.0% | 53 | 394 | 13.5% | 34 | 8.6% |  |  |  |  |
| Pathology characteristics | | | | | | | | | | | | | |
| Triple negative invasive* | 35 | 41.7% |  |  | 15 | 84 | 17.9% | 12 | 14.3% | 84.3 (74.0 – 90.8) | 79.9 (68.8 – 87.4) | 86.6 (76.4 – 92.5) | 83.5 (72.7 – 90.3) |
| Grade 3 ER+ HER2- # | 26 | 48.1% |  |  | 8 | 55 | 14.8% | 7 | 13.0% | 92.2 (80.4 – 97.0) | 86.9 (72.8 – 94.0) | 93.9 (82.3 -98.0) | 88.5 (74.3 – 95.1) |
| HER2+ | 9 | 60.0% |  |  | 1 | 15 | 6.7% | 1 | 6.7% | 90.9 (50.8-98.7) | - | 90.9 (50.8 – 98.7) | - |
| Lobular | 16 | 53.3% |  |  | 9 | 30 | 30.0% | 7 | 23.3% | 85.9 (66.7 – 94.5) | 81.2 (60.1 – 91.8) | 85.9 (66.7 – 94.5) | 85.9 (66.7 – 94.5) |
| Grade 2 ER+ | 36 | 37.2% |  |  | 11 | 95 | 10.6% | 4 | 4.3% | 94.7 (86.4 – 98.0) | 89.4 (78.9 – 94.9) | 98.7 (90.9 – 99.8) | 95.1 (85.3 – 98.4) |
| Grade 1 ER+ | 6 | 14.6% |  |  | 3 | 41 | 7.3% | 1 | 2.4% | 100 | 90.9 (68.3 – 97.7) | 100 | 95.5 (70.7 – 99.3) |
| Total invasive | 128 | 39.9% |  |  | 47 | 320 | 14.5% | 32 | 10.1% | 91.1 (87.0 – 93.8) | 85.7 (80.6 – 89.6) | 93.1 | (89.4 – 95.6) |
| CIS |  |  |  |  | 7 | 71 | 9.9% | 2 | 2.8% | 100 | 98.2 (87.6 – 99.7) | 100 | 98.2 (87.6 – 99.7) |

CIS: carcinoma *in situ*; RRM Risk Reducing Mastectomy; BC Breast cancer; ER+ Oestrogen receptor positive

*Includes 20 with no HER2 status presumed negative, #-Includes 13 Grade 3 ER+ with no HER2 status

**Supplementary table 2:** 20-year actuarial survival using 1-year intervals from dateofentry to dateofdeath or censoreddate for breast cancer specific deaths (cause of death Christie notes = BC)

All - 98.8 (98.3 – 99.1)

BRCA1 (n=365) – 96.1 (90.5 – 98.4)

BRCA2 (n=376) – 91.5 (78.6 – 96.8)
